# Startup process, safety and risk assessment of biomass gasification for off-grid rural electrification

**DOI:** 10.1038/s41598-023-46801-w

**Published:** 2023-12-04

**Authors:** Md Mashiur Rahman, Ulrik Birk Henriksen, Daniel Ciolkosz

**Affiliations:** 1 Department of Agricultural and Biological Engineering, The Pennsylvania State University, University Park, PA 16802 USA; 2 DTU Chemical Engineering, Technical University of Denmark (DTU), Frederiksborgvej 399, 4000 Roskilde, Denmark; 3 Agricultural Engineering Division, Pulses Research Center & Regional Agricultural Research Station, Bangladesh Agricultural Research Institute, Ishurdi, 6620 Pabna, Bangladesh

**Keywords:** Renewable energy, Bioenergy, Syngas

## Abstract

Biomass gasification has significantly advanced in terms of performance and is increasingly used in rural off-grid electricity applications. The downdraft gasifier is primarily used in biomass gasification applications, in which it functions as a reactor into which biomass and gasifying air are introduced to generate producer gas that is then used in an engine generator to produce electricity. However, the safety and stability of biomass gasification remain challenging and depend on several factors, such as the startup heating process, which can affect risks of fire, explosion, and toxic gas emissions. As the biomass gasification is associated with high temperatures and demands safety measures, its startup process should follow a rigorous procedure that ensures reliable operation and minimizes the risk of hazard issues. This study presents a gasifier startup heating process based on a proposed safety protocols hazard analysis. The study indicates that the heating temperature in startup processes has been identified as a critical factor due to its role in impacting safety. The findings indicate that the biomass gasification process has significant risks, including the potential for fire, explosion, and release of environmental emissions via multiple pathways. The methods proposed here could lead to reduced risk from the abovementioned issues.

## Introduction

### Importance of biomass gasification for off-grid rural electrification

Because of environmental concerns, reduced availability of oil and coal fuels and the growing problem of climate change, it is necessary to switch from fuels based on fossils to renewable energy sources. The need for new electrical power sources persists as a result of the growing use of electrically based technologies worldwide. Therefore, the development of new renewable electricity technologies is vital to provide for the long-term needs of society, taking into account the need for heightened reliability, security, and resilience in a nation's power system and the desire to reduce carbon emissions and air pollution resulting from energy production^1^. Due to this, the current global energy system is experiencing a shift from conventional fossil fuels to sustainable and renewable energy sources. This transition poses considerable technical obstacles, such as capital costs, required space, logistic problems, low efficiency and the need for human expertise to develop new technological remedies^2^. One promising approach is to install new decentralized power plants worldwide where there is a lack of accessibility to electricity from national electric grid systems.

Among all renewable energy sources, biomass-based energy, such as woody energy crops, agricultural crop residues, forestry residues and wood processing residues, is the primary renewable source of energy that is used significantly for a substantial number of rural and home-based businesses, sometimes known as "cottage industries," as well as the vast majority of rural families where electricity is unavailable. Biomass gasification is an affordable and promising renewable energy system that generates producer gas that can in turn be used in engine-based Combined Heat and Power (CHP) generation systems, among many other applications, for supplying thermal energy and generating electric power^2^. Currently, renewable fuels derived from biomass feedstocks account for less than 22 percent of the primary energy demand mix globally^1^. Primary forms of energy obtained from biomass are heat and electrical energy^3^. In a region with a relatively low population density and under geographical circumstances, it may not be economically feasible to supply the national electric grid to ensure electricity supply throughout the entire area. Therefore, off-grid technologies are necessary to meet the demands for electricity in these distant, unserved remote areas that are not currently supplied.

When compared to other off-grid renewable technologies, such as solar power, micro-hydro systems, wind turbine power, biogas, and micro-power, like LED lighting systems, biomass gasification is one of the most cost-effective options for standalone electricity generation in remote locations where the national electricity grid is not available^3, 4^. In order to provide these remote rural locations with electricity support, it is imperative that conventional oil-powered systems and the traditional, inefficient use of biomass be improved upon by introducing an efficient, decentralized source of biomass energy that can function as a sustainable source of heat and power at an affordable cost. By using biomass as a feedstock, the gasification process can transform biomass into producer gas comprised of CO, CO_2_, H_2_, CH_4_ and N_2_ by applying high temperatures combined with a limited supply of oxygen, gasifying air, steam, CO_2_ or a mix of them in a controlled environment^5, 6^. Making the producer gas relatively tar-free allows it to be used directly in an engine generator system to produce electricity and heat. Thus, if producer gas is intended to be utilized directly in an internal combustion engine (ICE), the most significant problem is the tar content of the producer gas^7, 8^. In rural off-grid areas where ICE is to be used, power generation demands the operation of a downdraft gasifier in order to generate producer gas characterized by a low-tar content, especially for downdraft throatless gasifiers, which is preferably a downdraft batch gasifier for producer gas generation for power applications^9, 10^. In order to operate biomass gasification systems efficiently, the understanding of the gasifier startup process and process-related safety measures and risk assessment should be used to implement a safety protocol for gasifier startup and to minimize the potential for hazardous effects on both human health and the environment.

### Importance of gasifier startup process, safety measures and risk assessment

Only a few research studies have discussed the gasifier startup process for coal gasification in large power plants, but it has not been studied for rural electrification in small-medium scale plants, nor has the startup heating process been described in detail from a process or safety perspective^11–15^. However, the identification and mitigation of safety issues are crucial for addressing the potential risks associated with explosion, fire, escape of poisonous tar, escape of toxic producer gas emissions, and operator’s failure. Considering these safety measures will allow for the identification of possible solutions to these safety challenges. The significance of gasifier startup for reactor heating and safety measures for assessing health and safety risks in the biomass gasification process during operation is of importance, as there is increased interest in using gasifiers for energy production. The need for more comprehensive risk evaluation due to the high temperature inside the gasifier during the startup process and shutdown holds significant importance, especially in the context of a novel and untested gasifier system. During the commissioning stage, these systems generally necessitate extended periods of operation, spanning from startup to shutdown. It is common for gasifiers to not progress beyond the commissioning stage^15^. Nevertheless, in the case of novel gasification systems that have not undergone testing before commercial applications, the time at startup and shutdown will be the primary focus, as expected during experimental operations.

The startup process for a gasifier is unique from steady operations in many respects. First, the temperatures within the gasifier change quickly as the gasifier is preheated. Furthermore, preheating initially occurs without biomass fuel in the gasifier, after which fuel is loaded into the reactor. This can cause dramatic variations in the temperature profiles, and flowrates and flow patterns of gases within the gasifier. In the authors’ observation, the potential risks associated with elevated temperatures are rarely acknowledged in commercial applications. Therefore, extra focus should be placed on startup, shutdown, and testing with an objective of mitigating harmful gas emissions and preventing safety hazards.

Moock and Trapp^11^ presents a study focusing on the startup process of coal gasifiers that utilize coal slurry and oxygen as the primary feedstock. During starting operations, it is necessary to engage in the flaring of raw gas until the commencement of downstream clean-up and recovery plants in order to ensure its readiness. Salen et al.^13^ states that the most significant risks of fire, explosion, and toxic gas occur during the starting up and shutting down of the gasification process or intermittent operation^12^. When the gasifier reactor fails to operate at its optimal capacity, the engine generator must stop the operation to safeguard both the reactor and other associated components from potential damage against dirty choking gas. In this cases, it immediately creates back pressure in the hot gasifier reactor, promptly releasing hazardous and flammable smoke^13^. Due to the gasifier's design, which is intended to operate at neutral pressure, any pressure generated during the shutdown stage results in gas leakage, even from gasifiers with a high level of tightness. Typically, during the startup of a gasifier installation, it is common practice to prevent the producer gases from passing through a gas filter. This precautionary measure is taken to prevent the filters from becoming blocked by the tars. In the downstream process, the filter retains air, and consequently, when an inflammable producer gas is generated and passes through the gas filter, there is a potential for the formation of an explosive mixture^14^. Reed and Das^15^ reports that the gasifier operates under sub-stoichiometric oxygen conditions, resulting in the condensation of tar and soot in the gasifier and gas outlet pipe due to organic acids in the water. Therefore, different zone heights of the gasifier, cooling system, and filtering system or anywhere tarry water condenses might cause the risk of tar deposition and corrosion. It is also observed that the reactor has the potential to undergo corrosion when exposed to elevated temperatures^15^.

The producer gas contains flammable components, mainly CO and H_2_ at concentrations of ca. 20 vol%, contributing to the potential hazards of fire, explosion, and gas leakage in the downstream processes^12^. Gas sensors and other system control techniques can reduce stand-alone gasifier emissions in these cases. Christiansen et al.^16^ reports safety issues related to operational practices and emissions reduction techniques employed in biomass gasification plants. Rollinson^12^ discusses many hazards like flammable, poisonous, and corrosive gas mixes, as well as the potential for explosions resulting from overpressure and heightened risk during startup, shutdown, or testing^12^. In order to ensure future safety by preventing risks, it is imperative to implement meticulous safety processes. Many safety assessment procedures have been developed and proposed for industrial processes, including Fault Tree Analysis, Hazard and Operability Analysis, Common Cause Failure Analysis, and Hazard Analysis and Critical Control Points^17^. These protocols all function to identify potential hazards in a system and develop methods for minimizing the likelihood of those hazards.

### Research challenges and objectives

The assessment of safety risks involving health and environmental issues during the startup heating of gasifier reactor and the biomass gasification process is a crucial step in reducing hazards associated with the processes. Furthermore, it aids in the development of optimal solutions to mitigate these risks in a manner that is convenient and efficient. Controlling the risk of fire, explosion, and harmful gas release from a gasification process requires a great deal of understanding, as the process is dependent not just on the design and operation of the reactor, but additionally to a great extent on the uniformity and variability of the biomass feedstock. Because of this, it is of the utmost importance to conduct safety risk assessments with great care.

Currently, there has not been any user-friendly methodology proposed for the biomass gasification startup heating process that addresses safety measures of these devices as well as health, risk and environment assessment for rural off-grid electrification. However, descriptive methodologies for the gasification startup process and safety measures for risk assessment are crucial needs for the widespread application of this technology for small to medium-scale rural off-grid electrifications. A lack of understanding of the heating process and limited awareness of the safety processes could result in neglecting environmental and health issues during lengthy and complex gasification startup procedures. Therefore, the challenge is to establish a gasification startup process that improves safety and reduces hazardous risks during the gasifier startup process. Such a guideline should be user friendly and be based on the accepted methodology, science, common sense, and wherever possible, measurable parameters during gasifier operation. For this, this proposed novel approach gives a user-friendly guideline for adopting the biomass gasification startup process and safety measures taken during operation to get a clean methodology for risk assessment.

## Methodology

In this study, a downdraft gasifier^7^ was started, operated and shut down to observe its process of operation. During this process, a hazard analysis was conducted, and safety protocols were developed for reduced hazard startup of gasifiers.

### Biomass gasification setup

**Figure 1:**
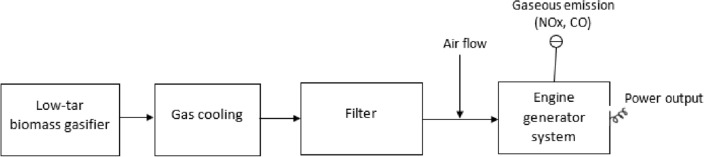
Simplified biomass gasification setup with an engine generator system to power output.

**Figure 2:**
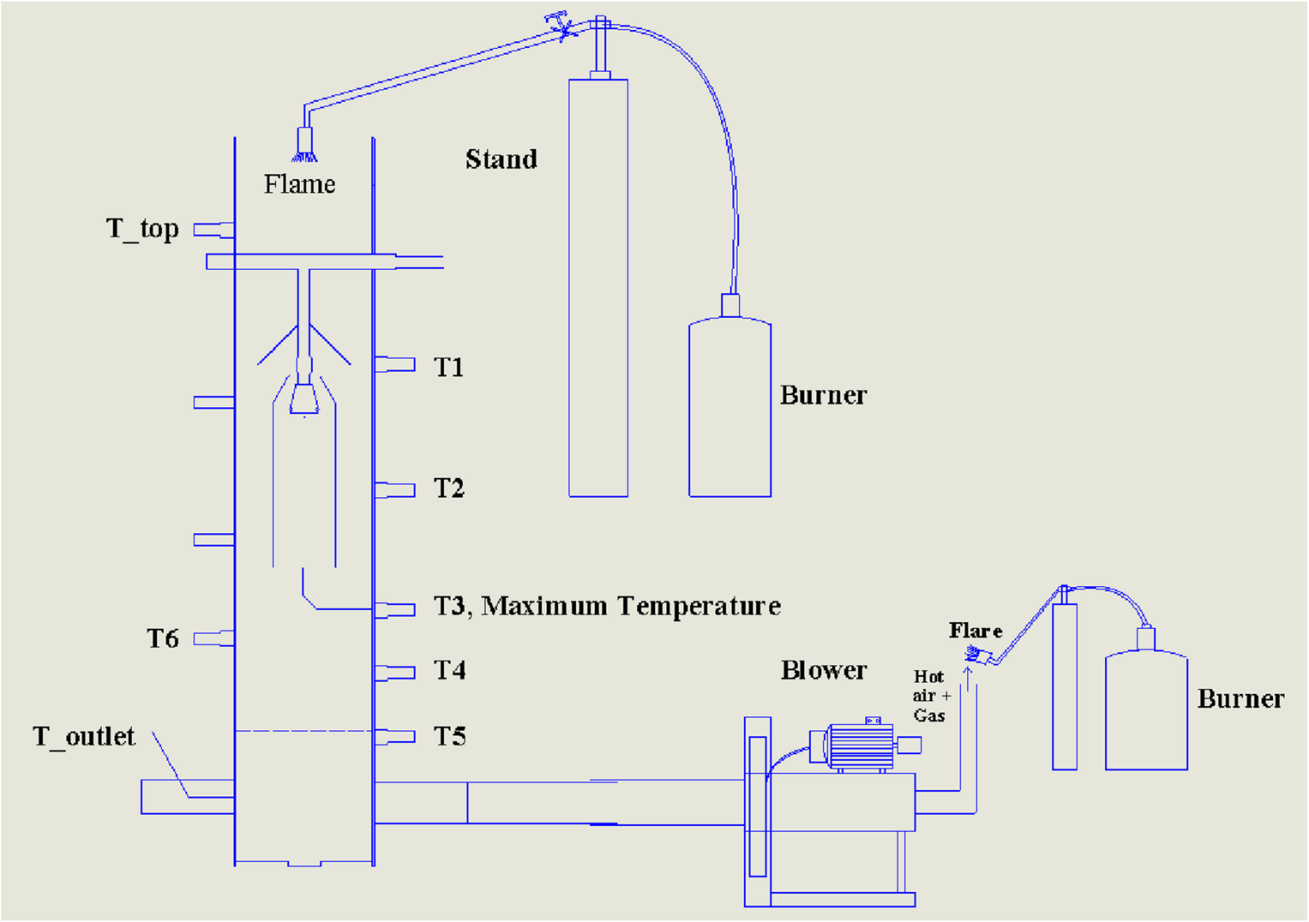
Schematic view of the biomass gasifier startup setup with the blower fan and LPG burner, T indicates the temperature sensor.

**Figure 3:**
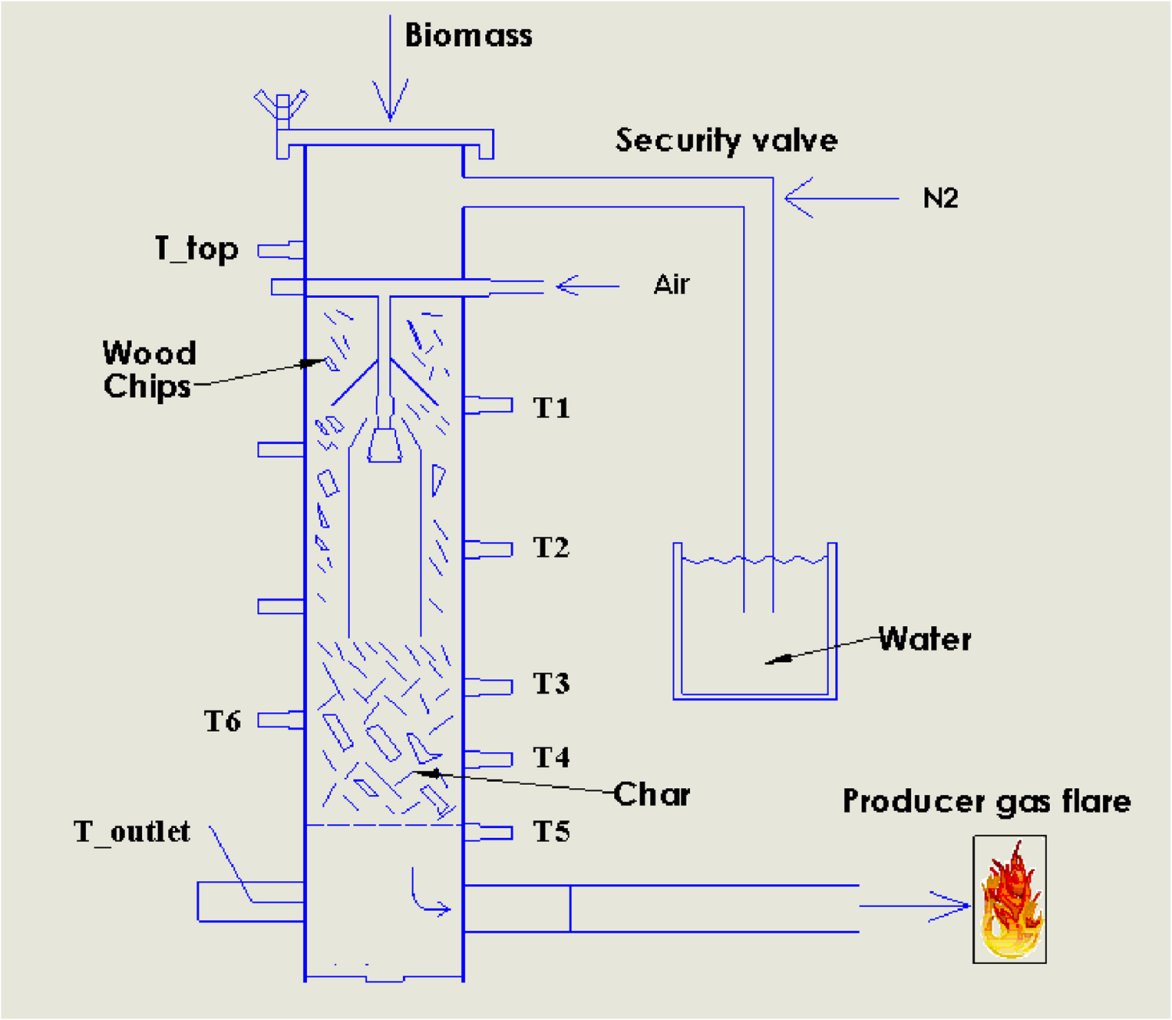
Schematic view of biomass gasifier startup heating setup with the security value, where T indicates the temperature sensor.

**Figure 4:**
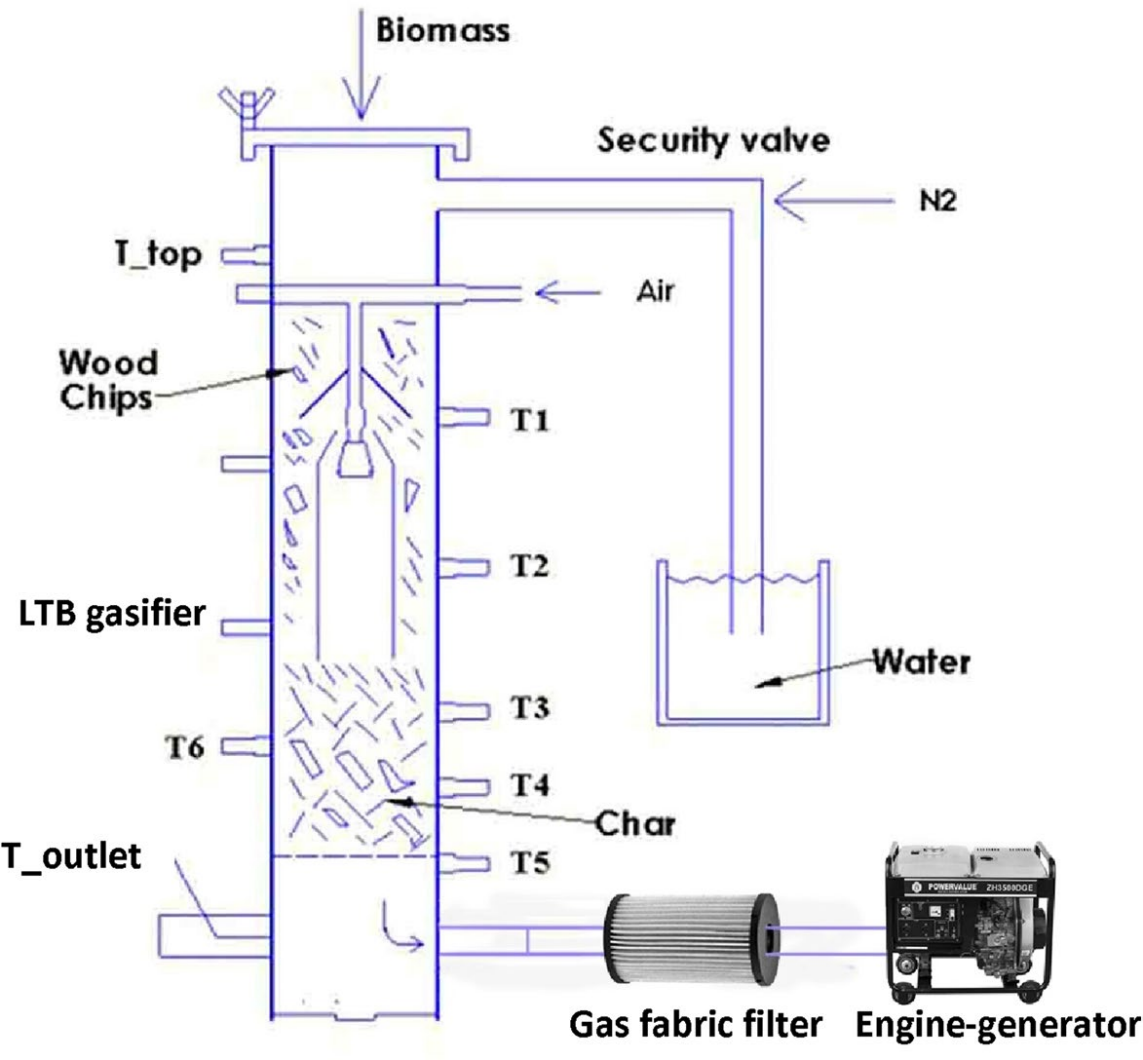
Schematic view of biomass gasification process setup with the LTB gasifier, gas filter and engine generator system, where T indicates the temperature sensor.

The biomass gasification setup consists of a low-tar biomass (LTB) gasifier, gasifier with an LPG burner, gasifier with a blower, gas cooling, gas filtration system, and an engine generator system, as shown in Fig. 1**.** The complete overview of the LTB gasifier and the setup process can be found in Refs.^7, 18, 19^. The LTB gasifier generates producer gas using biomass, which passes through a gas cooling system to cool the producer gas temperature, followed by a filter to remove impurities and particulate matter. The resulting clean producer gas is suitable for use in an engine generation system to produce electricity. The whole process has been described in detail in Refs.^7, 18, 19^. Figures 2 and 3 show the schematic views of the startup process for gasifier heating and biomass gasification process, respectively. Figure 2 shows the gasifier reactor, the LPG burner (O_2_ cylinder), long cooling pipe and blower fan during startup, while Fig. 4 depicts the gasifier reactor, security value with water, long cooling pipe, gas flare, gas filter and engine generator system during operation of the gasifier.

### Gasifier startup process and biomass gasification description

It is common to preheat the gasifier reactor to protect the integrity of the reactor lining both during gasifier startup and throughout the operation^11^. Typically, the preheating process necessitates using a relatively simple burner using methane or propane. The resulting byproducts of this combustion, namely carbon dioxide and water, are released into the atmosphere. After the preheating the gasifier, it is necessary to remove the gas burner in the reactor prior to gasifier startup; a certain amount of heat loss is unavoidable during this transition. The reactor is then started under ambient pressure, and the resulting producer gas from the reactor burns in a flare. Air is blown into the gasifier to start the gasification process. In the experimental operation, the gasifier heating startup process can be categorized into several steps, such as blower and liquefied petroleum gas (LPG) burner setup, warm-up process, operation process and shutdown process.

A simplified operation procedure can be divided by several steps and written as follows:

- **Blower and LPG burner setup:** Following the relocation of the gasifier reactor to an external location, the various sensor ports and equipment’s were examined correctly and sensor ports were connected to the computer data logger in order to record the data pertaining to the temperature and pressure. In the gasifier startup experiment, the reactor was positioned on a level surface and the LPG burner was placed at the top of the reactor and securely fixed to a stand near the reactor, as seen in Fig. 2. The producer gas outlet pipe of the reactor was connected to the blower fan before the gasifier heating process was carried out outside in an open space (Fig. 2)**.** An LPG burner was used to gradually heat the gasifier's reactor inside from the top and the blower was used so as to draw the hot heated air through the reactor (see Fig. 2). Before starting the gasifier warm-up process, the gasifier was operated without any biomass by turning on the blower to check the air leakage in the gasifier setup.
- **Warm-up Process:** After testing the leakage, the gas flow can be turned on and ignited, allowing heated air to be generated. Once the blower becomes operational, the resulting heated air gas (temperature ranges between 350 and 600 °C) inside the gasifier is consistently drawn through the system and discharged into the atmosphere through the producer gas outlet pipe. The hand-held gas burner (Bernz-O-Matic TS 7000) was used to ignite and allowed to burn the produced hot air gas. Once the different zones of the gasifier reactor reached a temperature between 800 and 900 °C, the blower was detached from the outlet pipe. The LPG burner and blower were then detached from the gasifier after it reached a steady temperature, as shown in Fig. 2.
- **Operational Process:** After detaching, the ignited hot char was loaded into the bottom of the reactor on the top of gasifier grate (reduction zone) to initial the gasification process. The reactor’s charbed was then filled with charcoal whose characteristics is same as char in available in market. Then, the rest of the reactor chamber was filled with biomass wood chips and the reactor top was securely fastened to the reactor. Pressurized air (6 bar) was then introduced into the reactor by turning on the valve of the air supply system and a flow meter was employed to assist in measuring the air supply rate, as well as to ensure that the correct amount of air was being supplied. After a few moments of time, it was seen that thick, white smoke was coming from the gas outlet pipe. This indicated that the producing gas was being produced and released. After a few minutes, clear producer gas was observed exiting the gasifier producer gas outlet. As the producer gas outlet temperature was observed at approximately 676 °C (avg.)^7^, it needed to be cooled down about 150 °C before going to the engine generator system. For this, the set up had a long pipe, that was sufficient to reduce the temperature of the producer gas and the temperature measuring sensor was used to measure the pipe outlet temperature to make sure the inlet temperature of the producer gas in the engine generator is below 150 °C. After that, the producer gas was put through a fabric filter to remove particulate matter. The cool producer gas was then ignited in a flare using a handheld torch (see Fig. 3). Instead of gasifier startup process while running the biomass gasification system to produce electricity (Fig. 4), the generated producer gas was conveyed through the ducting system of the engine generation system in order to produce electricity, while it is mixed with the air and combusted maintaining a proper air–fuel ratio within the ranges of 1.1–1.35 (see Fig. 1)^7^.
- **Shutdown Process:** At the close of the experiment, the gasifier heating process was shut down by using the gate valve to turn off the air supply into the gasifier, preventing more air from entering the gasification reactor. The control valve for the air supply system should be closed before turning off the pressurized air supply system. Once the pressurized air supply was switched off, the flow of nitrogen into the reactor was then activated to extinguish the flame quickly, resulting in rapid cooling and preventing the leftover char from burning (see Fig. 3). After that, the gasifier remained in an open area until it had cooled down completely and all of its gases had been released into the environment, a process that took more than 5 h.

During the biomass gasification operation, the gasifying airflow rate was regulated to achieve an optimum temperature, which corresponds to a high gasifier performance. In the LTB downdraft gasifier, the optimal combustion zone temperature was determined to be around 968 °C on average throughout the experiment of biomass gasification process when an air equivalence ratio of 0.27^7^.

## Results and discussion

### Temperature profile in the gasifier startup process

**Figure 5:**
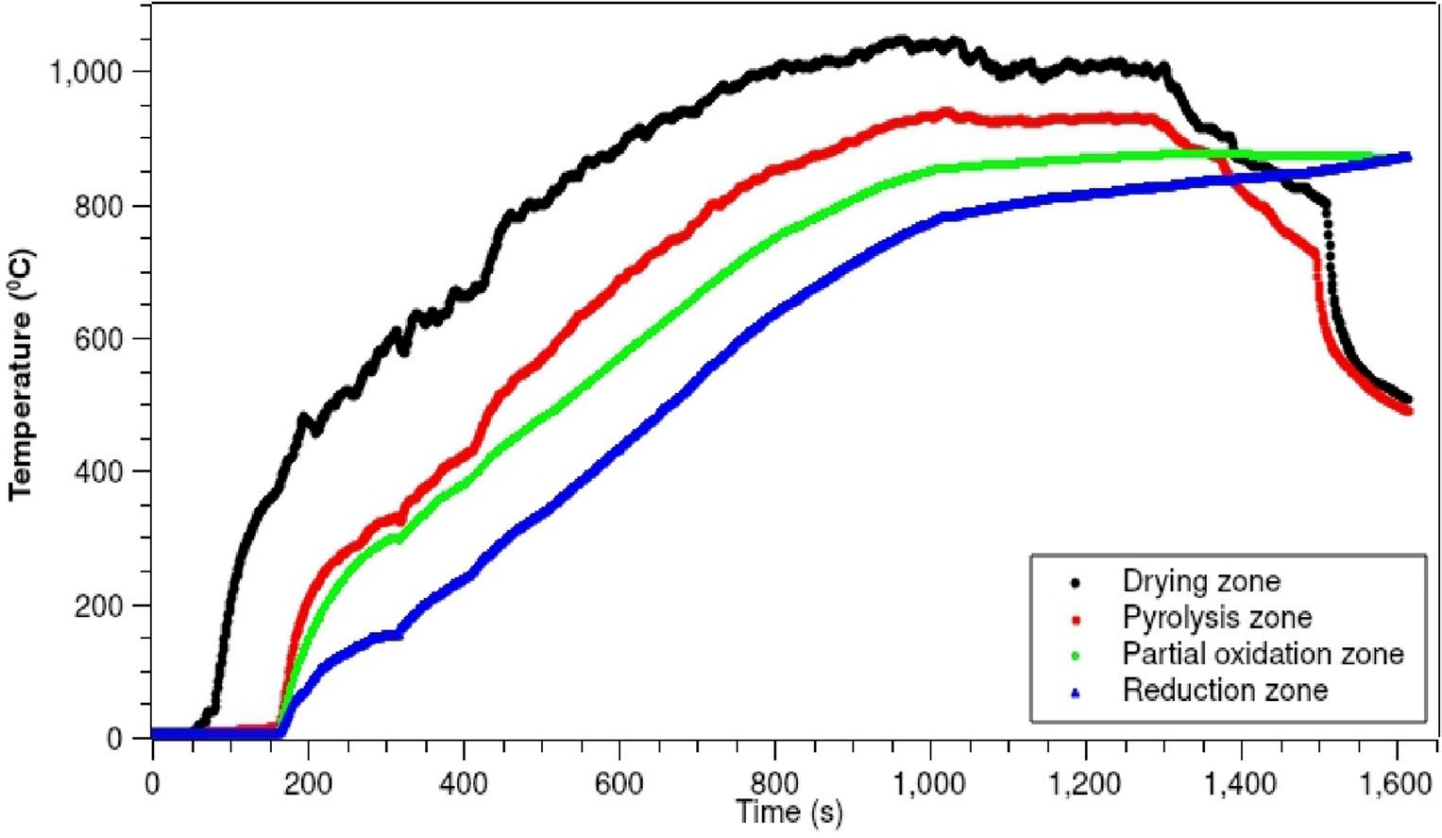
Typical gasifier temperature profile along the drying, pyrolysis, partial oxidation, and reduction zone during the gasification startup heating process.

Figure 5 shows time-series plots of the typical temperature profile in the drying zone, pyrolysis zone, partial oxidation zone and reduction zone as a function of time during the gasifier startup heating process. Understanding of this temperature in the startup heating process allows an operator to identify the point at which the gasifier is ready to begin biomass gasification operation. This plot underlines the potential to increase understanding of the proposed gasifier starting process by measuring the temperature ranges that occur as a result of different startup protocols and determining their impact on temperatures during operation of the gasifier. The temperature distribution inside the gasifier’s different zones will vary based on the position where the LPG burner output is placed for the preheating process. At the start of the heating process, the drying and pyrolysis zones of the gasifier experienced the highest temperature, which decreased gradually (Fig. 5). On the other hand, the temperatures in the partial oxidation and reduction zones increased slowly. The optimum time to initiate the biomass gasification occurs when the temperatures of different zones come together to a unique point, yielding a uniform temperature, or when the temperature gap between the partial oxidation and reduction zones approaches to zero, or when all temperature variations fall within a range of 100 °C. At the moment, the reactor is ready to take in biomass and initiate the process of biomass gasification inside the reactor.

### Hazard assessment issues in the biomass gasification startup process

Following the operation of the gasifier, a risk assessment was carried out in which potential hazards were identified, negative consequences of those hazards listed, and methods for minimizing the risk of those hazards were listed. As with any hazard assessment, this list is likely not yet complete, and may not apply to every gasifier configuration or operating protocol. Results of the assessment are shown in Table 1.

**Table 1:** Defining the key risk terms, and crucial safety risk assessment during the gasifier startup heating and biomass gasification process.

Main operational risks associated to the gasification startup setup			
Sl. No.	Risk	Unwanted consequences	Minimized by
1	Release of hot gases during startup process	CO, NOx, H_2_S emissions	A flare has the potential to burn off hot air
2	Temperature high in reactor (startup process)	Corrosion of reactor	The gas flow for the LPG burner is controlled manually by a valve to control reactor temperature. Reactor temperature will not exceed over 1000 °C because steel begins to soften and loses about half of its strength over 1000 °C
3	Temperature high in reactor (operating phase)	Material damage, leading to collapse of structure	The gasifying airflow rate for the gasification needs to be controlled to reduce the likelihood of gasifier materials damage
4		Burns to operators from contact with hot surfaces	All hot surfaces to be insulated, operators to wear appropriate PPE
5	Temperatue too low	Condensation of tar can occur in cold regions of the gasifier and gas outlet pipe	Nitrogen is blown into the gasifier, making a small high airflow rate and ensuring that all producer gas is going to the endpoint of the gasifier outlet
6	Pressure is too high	Explosion	Secured with a pressure release pipe in a water trap, as shown in Fig. 3. Pressure release mechanism included in case of explosion. Gasifier (including gas filter) inspected prior to use for buildup of residue, cleaned as needed
7	Leakage of toxic/combustible gases during operating process	CO, NOx emissions	Experiments are conducted in a open air, and a personal CO detector is worn at all times. If there was a leak, steps may be taken to fix it
8	Gas conditions become conducive for rapid combustion	Explosion	Draft fan used to control flow rate and flow direction of gases; gasifier sealed carefully to prevent uncontrolled injection of air. All equipment used in the area should be “spark proof”

### Safety measures during operation

Based on the hazard assessment, the following safety measures are proposed for startup of a biomass downdraft gasifier.

1. Gasification should be conducted outside in the open air or in a well-ventilated structure, and the flammable gases produced in the process should be immediately burned off with a flare or thermal oxidizer. The reactor’s outer surfaces should be covered with insulation materials (ceramic fiber insulation with R22 insulation) to reduce the risk of burns. The non-covered areas are the LPG heater and the producer gas outlet pipe, which should be clearly labelled as a burn hazard.
2. Before starting the gasification process, all the joining points, including the different sensor ports and equipment, should be checked and tightened as needed to avoid any leakage. In the case of the experiment carried out in this study, the top of the gasifier was tightened with the use of insulation materials (ceramic fiber insulation with R22 insulation) and was sealed well, preventing the leak of pyrolysis gases from the top of the drying zone during the biomass feedstock filling time.
3. The gas flow rate for the LPG burner should be regulated by a valve in order to manage the reactor temperature throughout the heating process. Moreover, this precautionary measure needs to be implemented to avoid an excessive increase in pressure drop inside the system. In order to mitigate this concern, a safety or security valve should be included as a preventive measure against potential explosions resulting from elevated temperatures and pressure (Fig. 3).
4. The use of hand gloves, safety shoes, and safety glasses should be mandatory throughout the process of putting biomass into the gasifier and working with the equipment. When the gasifier top is opened, a portion of the producing gas may escape from the drying zone of the gasifier. Hence, it is essential that the blower effectively extracts the producer gas from the reactor to prevent personnel exposure to the gas emitted by the gasifier.
5. During biomass fuelling of the gasifier, ideally, the blower fan will include a variable speed controller to maintain a steady pressure drop throughout the system. If the person filling the biomass into the gasifier smells smoke, they should be required to stop the filling and leave the gasifier immediately.
6. As the gasification setup is operated outside, a personal CO detector should always be worn by all personnel during the test. Upon detection of CO, operators should leave the area and shut down the system. After the system is fully cooled, a person can locate and repair the location from where gas emissions are occurring.
7. Operators should avoid looking into the ignition opening when introducing flame. The operator should use the computer data logger output to determine the condition of the temperature inside the reactor.
8. A safety valve should be included in the design in order to reduce the likelihood of accidental explosions from occurring as a result of higher pressure inside the gasifier. A “blow out panel” or similar explosion mitigation device should be included in the design, to reduce the severity of damage if an explosion does occur.

These guidelines are intended to be illustrative, showing the main safety hazards and methods that can be employed to mitigate those hazards. They can form the starting point for any operating gasifier’s startup safety protocol, including experimental gasifiers as well as commercial devices. The Supplementary Material document for this paper includes additional information pertaining to the safety assessment of experimental facilities.

## Concluding remarks

Biomass gasification offers significant advantages in terms of feedstock and outcome product versatility, as well as exceptional energy efficiency. It is an extremely important technological platform to evaluate in connection to renewable power generation. This study's goal was to introduce the concept of guidelines in the gasifier startup process for biomass gasification and operational safety measures. The gasifier startup process involves significant challenges, resulting in a high failure rate among small to medium-scale commercial biomass gasification systems. The times of startup and shutdown have been identified to be crucial moments during which there is a notably increased potential for fire, explosion, and emissions risks due to heightened temperature. The gasifier startup process results in the production of a hot gas that has significant flammability properties, as well as the release of toxic emissions. This is particularly concerning as it is essential for commissioning and functioning on a commercial basis while attempting to mitigate less risk hazards. The findings indicate that the biomass gasification process has significant risks including the potential for fire, explosion, and the release of environmental emissions via multiple pathways. The small to medium size biomass/wood gasifiers developed for small and micro power generation in integrated gas engines (internal combustion engine, Rankine cycle and gas generator) could operate more successfully in rural off-grid locations with the successful implementation of the startup safety protocol outlined here. Future studies of gasifier startup process and safety measure for the biomass gasification process could include further study of the links between temperature and pressure when the system is being tested, as well as establishment of fail-safe thresholds to be used for automated shutdown when the system is not operating as expected.

### Supplementary Information

Supplementary Information.

## Data Availability

The datasets used in this study may be obtained from the corresponding author upon a reasonable request.
