# Supplementary material for: Startup process, safety and risk assessment of biomass gasification for off-grid rural electrification
Source: Sci Rep. 2023 Dec 4;13:21395. doi: 10.1038/s41598-023-46801-w (PMC10696013; doi:10.1038/s41598-023-46801-w)
Supplement: Supplementary file 1 — Supplementary Information. [file 41598_2023_46801_MOESM1_ESM.docx]

## Supplementary Materials : Safety Assessment of experimental facilities

| **Setup Identification** | | | | |
| --- | --- | --- | --- | --- |
| Name of the setup | | **Low-tar Biomass (LTB) Gasifier Unit** | | |
| Location | | Next to building 321, Risø campus, Frederiksborgvej 399, 4000 Roskilde | | |
| Research group | | Biomass Gasification Group (BGG) | | |
| Project manager | | Ulrik Birk Henriksen, Jesper Ahrenfeldt, Maria Puig Arnavat | | |
| Safety assessment carried out by | | Md Mashiur Rahman | Date: | 14/3/2018 |
| Local safety group | Signature |  | Date: |  |
|  | Signature |  | Date: |  |
| Control | Signature |  | Date: |  |

| **Description** | |
| --- | --- |
| Purpose | The setup is used for gasification of biomass at high temperatures. The purpose of the setup is to study the characterization of gas composition of producer gas and tar content using wood pellets and wood chips by thermochemical process. The setup is also used to evaluate mass and energy balance to figure out gasifier capacity, gasification efficiency, energy efficiency and optimization of operation parameters. |
| **Setup Description:**  The gasification tests are carried out outdoors. Wood chips with 32.28% moisture content are filled up into the gasifier. The gasifier reactor is heated externally by an LPG burner and gasifier is heated up gradually. The blower connected to the producer gas outlet pipe is turned on and hot air is sucked continuously and discharges it into the atmosphere. Once the gasifier is heated at 800^o^C-900^o^C, the blower is detached from the outlet pipe. The biomass is filled up into the gasifier and the gasifier top is fitted tightly with the reactor. The released producer gases are burned afterwards by the flare. Figure 1 shows the Gasifier setup.  A simplified operation procedure can be written as:   - **Blower and LPG burner setup:** After placing the gasifier reactor outside, blower is set up in the right place, and it can be turned on to suck hot air from the reactor. The LPG burner is placed near the reactor and is fixed with a stand. The gas flow can be turned on and LPG burner can be ignited. - **Warm-up:** The LPG burner is ignited with a hand-held gas burner (Bernz-O-Matic TS 7000) and gasifier reactor is heated at 800^o^C-900^o^C. After reaching a stable temperature, LPF burner and blower are detached from the gasifier which is represented by figure 1(a). The char bed of gasifier is filled up with glowing char and after few moments fresh cold char is put into the reactor char bed. Then the whole reactor is filled up with biomass. The valve of the air supply system is then turned on to supply pressurized air into the reactor. Gasifier temperature and pressure difference at different zones are monitored continuously with the help of thermocouple (T1-T6, T_top and T_outlet) and U-tube manometer, respectively. - **Producer Gas:** The producer gas is produced immediately, and the producer gas is ignited by the torch which is represented by figure 1(b). - **Shut down:** Stopping the following order: 1) turned off the valve of air supply system to prior to cutting off the pressurized air, 2) turn on the value of N_2_ supply (see figure 1(b)) in to the reactor.  \| 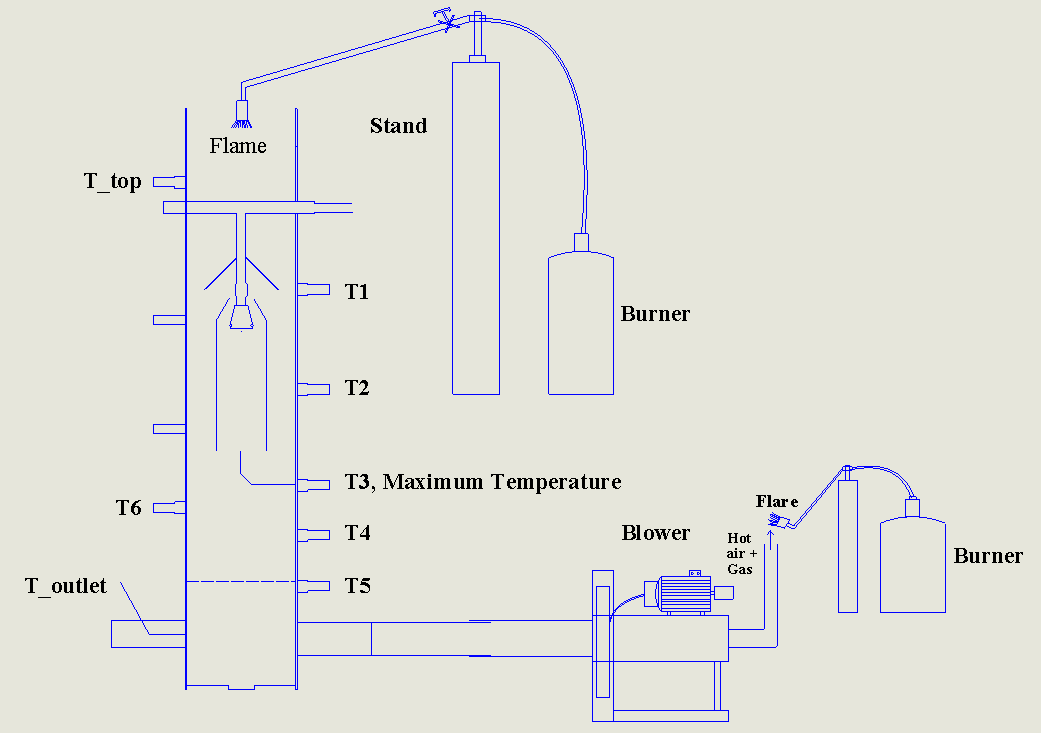 \| \| --- \| \| Figure F.1: Gasifier setup with the blower and burner. \| \| 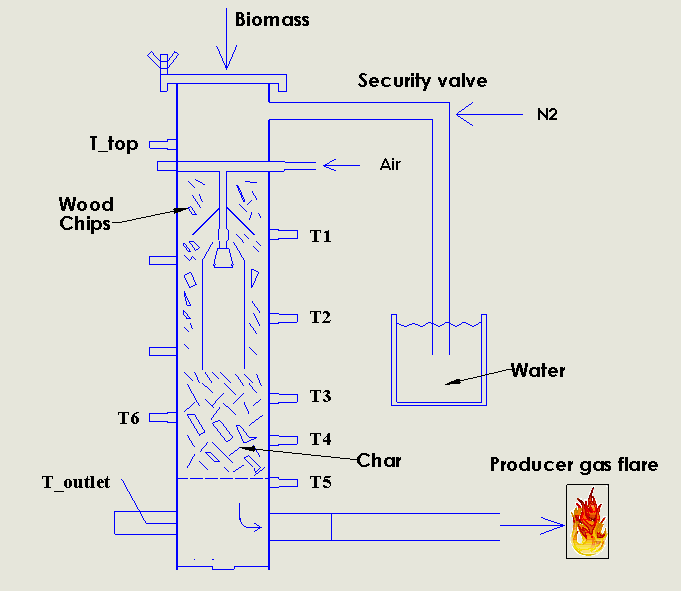  Figure F.2: Gasifier setup with combustion of producer gas. \|   **Safety mechanisms:**   - The reactor is covered in isolated materials which prevent personal damage. The non-covered areas are the flame of the LPG heater, pipe of producer gas outlet and flame of producer gas. - The personnel always wear hand gloves, safety shoes and safety glasses when filling the biomass in to the gasifier. - The blower is sucking from the gas outlet pipe. The blower has a VLT, and personnel are adjusting the rotation speed so that the suction is just enough to avoid smoke rising against the person filling biomass in the gasifier. If the person filling the biomass with the gasifier smells smoke, he will stop the filling and leave the gasifier immediately. - The setup is operated outside, and a personal CO detector is worn always during the test. - The gas flow for the LPG burner is controlled manually by a valve to control temperatures of reactor. Reactor temperature will not exceed over 1000°C because steel begins to soften and loses about half of its strength over 1000°C. - The top of the gasifier is tightened with the top cover by well-sealed materials, so it prevents the leak of toxic pyrolysis gases. - Security valve is installed to the top of gasifier so that it makes sure to avoid in any accidental problems. | |

| **Operating conditions and main specifications** | |
| --- | --- |
| Reactor or main vessel size | Length – 1475 mm, inside diameter – 256 mm |
| Normal operating temp/max temp | 900⁰C/1000^o^C |
| Normal operating pressure/max pressure | Atmospheric pressure |
| Other relevant specifications |  |

| **Discharge to environment from Materials and Chemicals involved** | | | |
| --- | --- | --- | --- |
|  | Component(s) | Amounts –  discharged to where? | CHEM-APV  approved? |
| Gases | CO | After combustion of producer CO gas is released. H_2_0, CO_2_ are released from the flare |  |
| Liquids | Tar, H_2_O | H_2_0 is vaporized and tar is combusted in the flare |  |
| Solids | Ash, biochar | Biochar and ash are stored afterwards in sealed polybags. The biochar is not toxic |  |
| Dust |  |  |  |
| Odors |  |  |  |
| **Consumption from building supply** |  |  |  |
| Gases | LPG | LPG is used for heating the gasifier |  |
| Liquids |  |  |  |
| Solids | Wood Pellets, Wood Chips | Biomass is stored in closed barrels prior to gasification. |  |
| Electrical power | Motor | Power is supplied to operate the blower |  |

| **Main operational risks associated to the setup**  Please describe the main risks of this setup and what has been done to minimize these risks. Use” Analysis of deviations from normal operation” below, as a work tool to identify and take measures against risks. | | |
| --- | --- | --- |
|  | **Risk** | **Minimized by** |
| 1 | Explosion | The gas flow for the LPG burner is controlled manually by a valve to control temperatures of reactor. Reactor temperature will not exceed over 1000°C because steel begins to soften and loses about half of its strength over 1000 C. |
| 2 | Leakage of toxic/combustible gases | The setup is operated outside, and a personal CO detector is worn always during experiments. |
| 3 | High temperature | Most hot elements are insulated to avoid personal damage (except the LPG burner flame and torch). |
| 4 | Moving parts |  |

**When the Safety Assessment is completed and acknowledged by the local safety group, please send it to the KT safety manager including relevant appendices**

**Analysis of deviations from normal operation**

Use the following form as a tool to identify and evaluate risks. Add elements if necessary.

| **Setup:**  **Section:** | | **Person:** | **Date:** |
| --- | --- | --- | --- |
| **A. Problem/deviation** | **B. Unwanted consequences of deviation** | **C. Measures taken to avoid unwanted consequences** | **D. Further actions to be taken to reach an acceptable situation** |
| **1.Flow** |  |  |  |
| 1.1.Gas |  |  |  |
| *1.1.1.Too much* |  |  |  |
| *1.1.2. Too little* |  |  |  |
| 1.2. Fluids |  |  |  |
| *1.2.1.Too much* |  |  |  |
| *1.2.2. Too little* |  |  |  |
| **3.Temperature** |  |  |  |
| 3.1 Gas in reactor |  |  |  |
| *3.1.1. Too high* | Material damage | Thermocouples are installed to monitor the temperatures in the gasifier. The gasification process is always monitored by personal. |  |
| *3.1.2.Too low* |  |  |  |
| 3.2 . Fluids |  |  |  |
| 3.2.1 *Too high* |  |  |  |
| 3.2.2. *Too low* | Condensation of tar can occur in cold regions of the gasifier and gas outlet pipe | Nitrogen is blown in to the gasifier, making a small high-pressure and ensuring that all producer gas is going to the endpoint of the outlet. | Always use gloves (work gloves/nitrile) when detaching the biochar/ash from the gasifier after end run, to avoid skin contact with tar. |
| **4. Pressure** |  |  |  |
| 4.1. Gas in reactor |  |  |  |
| *4.1.1. Too high* | Explosion | Pressure too high - secured with pressure release pipe in a water trap. |  |
| *4.1.2. Too low* |  |  |  |
| 4.2. Fluids |  |  |  |
| *4.2.1 Too high* |  |  |  |
| *4.2.2. Too low* |  |  |  |
| **5.Materials/chemicals** (The considerations on materials can also be dealt with on APV’s) | | | |
| 5.1. Poisonous, Radioactive  or carcinogenic |  |  |  |
| 5.2. Handling | Inhaling dust when biomass is loaded | A filter mask is always used when loading biomass. |  |
| 5.3. Leakage | CO, NO_x_ leakage | The setup is only used outside, and a personal CO gas alarm is worn always. Cool down of gasifier is also outside. |  |
| 5.4. Disposal |  |  |  |
|  |  |  |  |
|  |  |  |  |
| **6. Ventilation** |  |  |  |
| 6.1. Cut-off |  |  |  |
| 6.2 Reappearance |  |  |  |
| **7. Electrical power** |  |  |  |
| *7.1. Cut-off* | If the blower stops during loading, the person who loads the reactor can be exposed to harmful emission | There will always be a person present to operate the blower, when the reactor is loaded, and can therefore warn if the blower stops. |  |
| *7.2. Reappearance* |  |  |  |
| **8. Mechanical** |  |  |  |
| ? |  |  |  |
| ? |  |  |  |
| ? |  |  |  |
